# Perceived usefulness of COVID-19 tools for contact tracing among contact tracers in Korea

**DOI:** 10.4178/epih.e2022106

**Published:** 2022-11-15

**Authors:** Seonyeong Gong, Jong Youn Moon, Jaehun Jung

**Affiliations:** 1 Department of Preventive Medicine, Gachon University College of Medicine, Incheon, Korea; 2 Artificial Intelligence and Big-Data Convergence Center, Gil Medical Center, Gachon University College of Medicine, Incheon, Korea; 3 Center for Public Healthcare, Gachon University Gil Medical Center, Incheon, Korea

**Keywords:** COVID-19, Contact tracing, Epidemiology, Communicable diseases

## Abstract

**OBJECTIVES:**

In Korea, contact tracing for coronavirus disease 2019 is conducted using information from credit card records, handwritten visitor logs, KI-Pass (QR code), and the Safe Call system after an interview. We investigated the usefulness of these tools for contact tracing.

**METHODS:**

An anonymous survey was conducted for 2 months (July to September 2021) among contact tracers throughout Korea. The questionnaire consisted of 4 parts: (1) demographic characteristics; (2) the usefulness of each tool for contact tracing; (3) the order in which information was checked during contact tracing; and (4) the match rate between tools for contact tracing, screening test rate, response rate, and helpfulness (rated on a Likert scale).

**RESULTS:**

In total, 190 individuals completed the survey. When asked to rate the usefulness of each tool for contact tracing on a Likert scale, most respondents (86.3%) provided positive responses for credit card records, while the most common responses for handwritten visitor logs were negative. The highest percentage of positive responses for helpfulness was found for KI-Pass (91.1%), followed in descending order by credit card records (82.6%), Safe Call (78.2%), and handwritten visitor logs (22.1%).

**CONCLUSIONS:**

Over 80% of participants provided positive responses for credit card records, KI-Pass, and Safe Call data, while approximately 50% provided negative responses regarding the usefulness of handwritten visitor logs. Our findings highlight the need to unify systems for post-interview contact tracing to increase their convenience for contact tracers, as well as the need to improve tools utilizing handwritten visitor logs for digitally vulnerable groups.

## GRAPHICAL ABSTRACT

[Fig f1-epih-44-e2022106]

## INTRODUCTION

Coronavirus disease 2019 (COVID-19) has caused significant damage in countries worldwide, including Korea. Based on the lessons learned from the Middle East respiratory syndrome outbreak in 2015, Korea implemented the use of objectively verifiable information, such as credit card records, closed-circuit television (CCTV) footage, global positioning system (GPS) data, and medical facility information, for more timely identification of contacts during the COVID-19 pandemic [1]. In Korea, epidemiological investigations begin with an interview (telephone, face-to-face, or written) [2]. However, these interviews are limited in that they rely on the collection of subjective statements, which are vulnerable to uncertain memories, false statements, and omission and concealment of information. To address this problem, some objective information may be used during contact tracing such as GPS data, credit card information, CCTV footage, and drug utilization reviews (Table 1) [2].

Since 2020, it has been recommended that vulnerable facilities in Korea should implement visitor logs, but loopholes in handwritten visitor logs have been revealed in the investigation of an outbreak at nightclubs in Itaewon [3,4]. In June 2020, the Ministry of Health and Welfare developed and implemented KI-Pass, a QR code-based electronic entry log system, to improve upon handwritten entry logs, which may be vulnerable to false reporting and information leakage [4]. KI-Pass is used in the following manner. When someone uses a facility, a major social network service company in Korea (e.g., Naver, Kakao Talk, or PASS) issues a personal QR code to that individual. The code is presented to the facility manager, who then scans it to generate a record of the visit. If a case is later confirmed at that facility, health authorities including the Korean Disease Control and Prevention Agency (KDCA) request personal information from the QR code-issuing company (name, telephone number, and QR code) and Korea Social Security Information Service (facility, time of visit, and QR code). The information obtained through this process is combined for use in implementing infectious disease control measures, including contact tracing. The stored information is automatically destroyed after 4 weeks [4].

In September 2020, the city of Goyang made public the Safe Call Access Management System, which assigns a unique Safe Call number to each business that can be used to interact with and notify customers before their visit [5]. All Safe Call records are stored on a separate server. This system is advantageous in that there is little concern regarding the infringement of personal information, it is easy to operate, and it enables the timely identification of contacts since contact tracers can directly access the system to verify each visit. As with KI-Pass data, the stored information is automatically destroyed after 4 weeks [4].

However, the usefulness of these tools for contact tracing to complement interviews in epidemiological investigations has not been studied. This study aimed to evaluate the usefulness of various tools for contact tracing by conducting an online survey of contact tracers.

## MATERIALS AND METHODS

We developed a survey targeting contact tracers in Korea who had experience performing contact tracing of confirmed COVID19 cases. The total number of potential participants was estimated to be approximately 1,800 individuals, including 155 specialists added to 5 regional centers in 2020 for disease control and prevention and 1,066 contact tracers designated by local governments. To recruit participants, the link to the online questionnaire was sent to the heads of departments in charge of contact tracing throughout Korea via email. The online survey was conducted from July to September 2021 using Google Forms. The survey was conducted anonymously, and all respondents participated voluntarily. Due to the revision of some questions during the survey period, the number of responses differed for some questions (The survey in the Korean language is available in Supplementary Material 1).

The questionnaire used in the current study consisted of the following four parts:

(1) Demographic characteristics (sex, age, affiliation, position, working period, and location)

The possible affiliations included higher-level units of local government, basic units of local government, Communicable Diseases Centers, and the KDCA, and the positions were divided into public health doctors and officers. Working experience related to infectious disease was classified as < 1 year, 1-3 years, 3-10 years, and ≥ 10 years. The location was classified as the capital area, non-capital areas, and KDCA.

(2) The usefulness of each tool for contact tracing (rated on a Likert scale)

For each contact tracing tool, participants were asked to choose a response from 1 to 5 for their perceived usefulness, with 1 being the most negative answer.

(3) Order in which information was checked during contact tracing

We asked respondents to indicate the first tool that they used to check information after an interview.

(4) Match between tools for contact tracing, screening test rate, response rate, and helpfulness (rated on a Likert scale)

For each contact tracing tool, we asked participants to respond from 1 to 5 depending on their perceptions of how much the information from a given tool matched the actual information (information match rate), how many people performed screening tests (screening test rate), how many people they were able to contact (response rate), and how helpful a tool was to the overall epidemiological investigation (helpfulness). The most negative answer was 1.

A descriptive statistical analysis of the survey results was performed using R version 4.1.1 (R Core Team, Vienna, Austria) and Excel 2016 (Microsoft Corp., Redmond, WA, USA).

### Ethics statement

This study was approved by the Institutional Review Board of the Gachon University Gil Medical Center (protocol GFIRB2021-217), which waived the need for informed content. All study methods were carried out based on the Declaration of Helsinki.

## RESULTS

### Participant characteristics

In total, 190 individuals participated in the survey. Assuming that the total number of contact tracers in Korea was 1,800, the response rate was 10.5%. The demographic characteristics of the respondents are shown in Table 2. The respondents included 75 males (39.5%) and 115 females (60.5%). When categorized into age groups, there were 61 (32.1%), 82 (43.2%), 33 (17.4%), 12 (6.3%), and 2 (1.1%) individuals aged 20-29 years, 30-39 years, 40-49 years, 50-59 years, and ≥ 60 years, respectively. Most participants responded that they were affiliated with basic units of local government (n=139, 73.2%), followed by higher levels of local government (n=31, 16.3%), the KDCA (n=13, 6.8%), and Communicable Diseases Centers (n=7, 3.7%). The most common location was the capital area (n=148, 77.9%), followed by non-capital areas (n=29, 15.3%) and the KDCA (n=13, 6.8%).

### Usefulness of each tool

From the usefulness of each tool for contact tracing on a Likert scale, most respondents (86.3%) provided positive responses for credit card records, followed by KI-Pass (QR code) (83.2%), GPS and CCTV (80.5%), a statement from the confirmed patient or facility manager (68.9%), Safe Call (64.2%), sending a disaster message (39.5%), and handwritten visitor logs (21.1%) (Table 3).

Eighty-nine percent of respondents indicated that the KI-Pass (QR code) data matched the actual visitor information. In addition, 74.2% of respondents indicated that screening tests were performed among contacts identified using KI-Pass (QR code) information. Similarly, 80.5% of respondents indicated that individuals could be contacted using KI-Pass (QR code) information. In terms of overall helpfulness, 91.1% of respondents indicated that KI-Pass (QR code) information was useful for contact tracing (Table 4).

Eighty-eight percent of respondents reported that the information from credit card records matched the actual visitor information, while 73.2% noted that screening tests were performed among individuals contacted based on credit card records. Similarly, 78.4% of respondents indicated that individuals could be contacted using credit card information. In terms of overall helpfulness, 82.6% of respondents indicated that credit card records are useful for contact tracing (Table 4).

Eighty-six percent of respondents indicated that the Safe Call information matched the actual visitor information, while 74.4% noted that screening tests were performed among individuals contacted based on Safe Call information. Similarly, 75.2% of respondents indicated that individuals could be contacted using Safe Call information. In terms of overall helpfulness, 78.2% of respondents indicated that Safe Call information was useful for contact tracing (Table 4).

However, only 15.8% of respondents stated that the information from handwritten visitor logs matched the actual visitor information, while 27.9% noted that screening tests were performed among individuals contacted based on handwritten visitor logs. Similarly, 29.5% of respondents indicated that individuals could be contacted using the information in handwritten visitor logs. In terms of overall helpfulness, 22.1% of respondents indicated that handwritten visitor logs were useful for contact tracing (Table 4).

### Order of checking sources of information during contact tracing

Credit card records were most commonly identified as the first source of information checked during contact tracing (40.0%). The most common response for the second source of information checked was KI-Pass (QR code) (31.1%), followed closely by GPS data and CCTV footage (30.0%). KI-Pass (QR code) was also the most common response for the third source of information checked (35.3%), while Safe Call was the most common response for the fourth source of information checked (28.9%).

In contrast, when asked to indicate the first source of information checked during contact tracing for a mass outbreak, KI-Pass (QR code) was the most common response for both the first and second sources of information. Handwritten visitor logs represented the most common response for the third source of information checked (28.5%), while Safe Call data represented the most common response for the fourth source of information checked (25.9%) (Table 5).

## DISCUSSION

In the present study, we conducted an online survey of actual contact tracers to assess the usefulness of various tools for contact tracing. Credit card records were regarded as the source of information with the highest usefulness for contact tracing. Moreover, 87.9% of participants provided a positive response when asked whether the data from credit card records matched the actual visitor information. According to a 2019 survey by the Bank of Korea, credit cards were the most popular form of payment among Korean consumers. The popularity of credit card payments has increased in recent years, which can be explained by its perceived convenience [7]. Accordingly, the perceived usefulness of credit card records for contact tracing may have been the highest among the tools investigated because most people use credit cards for payment, and credit card transactions leave records similar to entry logs and allow the definitive identification of users. Moreover, to aid in the timely tracing of contacts and infection sources, the Ministry of Land, Infrastructure, and Transport developed the Epidemic Investigation Support System (EISS), which has been operated by KDCA since March 26, 2020. the EISS, which is currently operating, expedites contact tracing by collecting credit card transaction records in real-time and uses that information to conduct temporo-spatial analysis [8].

The tool for contact tracing that was assessed to have high usefulness by the second-highest percentage of respondents was KI-Pass (QR code). In 2021, the smartphone usage rate among Korean adults was 94.7% [9], meaning that almost all adults utilized smartphones. The frequency of positive responses regarding the high usefulness of KI-Pass (QR code) is understandable, as anyone with a smartphone can use this convenient system to fill out an entry log, allowing for accurate records of most visits. As shown in Table 5, there was a difference in the information they checked first according to whether or not contact tracing as performed in the context of a mass outbreak. A disadvantage of using credit card records is that usually only one person from a group uses his or her credit card to pay, necessitating the cumbersome step of having to ask the payer for information regarding other members of the party. However, because KI-Pass can be used to check each person who owns a smartphone, this issue can be avoided.

In contrast, handwritten visitor logs were the tool that the highest percentage of respondents assessed as having low usefulness. This may be because most businesses do not manage their handwritten visitor logs very well, meaning that the information may be false, missing, or at risk for leakage. In the case of the mass outbreak in 2020 involving a club in Itaewon, only 41% of names appearing in the handwritten visitor log could be contacted [4]. Despite guidelines stipulating that the logs must be managed so that others cannot view them, there have been numerous cases concerning the illegal use of personal information contained in handwritten visitor logs from businesses that did not follow such guidelines [10]. To protect personal information appearing in handwritten visitor logs, the Korean government implemented a “personal safe number” [11] that could be used instead of a mobile phone number. However, because this “personal safe number” can be obtained through private companies, digitally vulnerable groups still face difficulties. Moreover, only one person from a group may fill out the log, handwriting may be illegible, and there is concern regarding the risk of infection due to the sharing of writing instruments. Despite these issues, handwritten visitor logs cannot be eliminated, as digitally vulnerable groups and people who have lost or left their phones at home would not otherwise be able to fill out an entry log. Therefore, tools for completing and managing handwritten visitor logs should be improved; possible options might involve the use of a digital kiosk or an electronic device, such as a tablet PC.

In other countries, COVID-19 contact tracing is conducted based on statements from confirmed patients obtained through interviews. Some countries, including the United States, England, Australia, Japan, Italy, and Germany, are investigating the routes of movement and contacts of confirmed patients with COVID-19 using telephone, text messaging, and email records. Additionally, a Bluetooth-based contact tracing app can be used to identify the routes of movement as well as contacts that a person is unable to recall [12]. A user can download this app to his or her smartphone, and nearby devices share anonymous IDs when Bluetooth is activated. Subsequently, if a confirmed case occurs among shared IDs, then a notification is sent to each user’s smartphone. However, the identity of the confirmed patient is not revealed. This app is advantageous for protecting the personal information of users since it relies only on Bluetooth functionality rather than personal information such as name and location. However, using this app is optional, meaning that it would not be of significant help to infectious disease control efforts if the app installation rate is low. Even if a notification of being in contact with a confirmed patient is received, undergoing a screening test is only recommended, not required. Therefore, if an infected recipient ignores the notification, a mass outbreak may occur. Contact tracing apps are also not applicable for digitally vulnerable groups.

The present study had some limitations. First, only 190 contact tracers participated in the survey, and the findings may not be valid in representing all contact tracers in Korea. Furthermore, since only 190 out of 1,800 contact tracers participated in the survey, the response rate was only 10.5%. At the time of the survey, Korea had an average of more than 1,000 confirmed COVID-19 cases per day, so it was a busy time for contact tracers in Korea. Second, the validity and reliability of the questionnaire used in this study have not been verified in previous studies. However, when 190 out of 1,800 respondents responded, a 7% margin of error was calculated with a 95% confidence level. Therefore, most of the results of this study can be interpreted as meaningful results despite the low response rate.

Our survey results highlight the need to develop a system that can compensate for the weak points of handwritten visitor logs, such as one that utilizes digital devices supplied by businesses. Moreover, the amount of information that contact tracers must assess during contact tracing has increased due to the development of various types of entry log systems. This can lead to increased fatigue and interfere with effective contact tracing. Therefore, the present findings suggest the need to develop an efficient entry log system for managing and responding to emerging infectious diseases in the COVID-19 era.

## Figures and Tables

**Figure f1-epih-44-e2022106:**
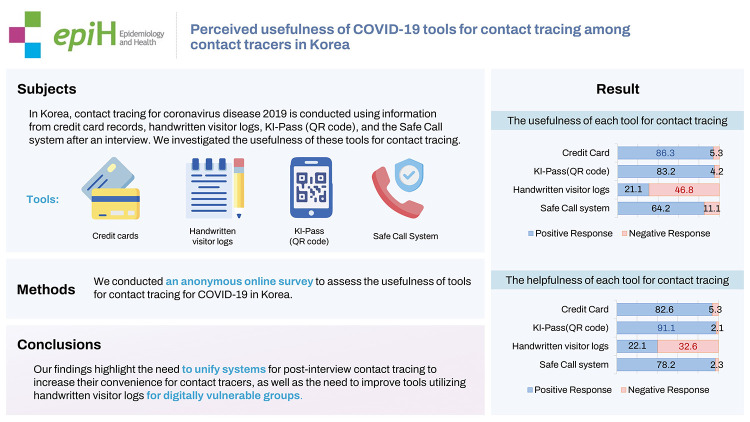


**Table 1.** Detailed procedures for COVID-19 contact tracing in Korea (as of March 2021)

Category	Item	Details	Responsible parties
Stage 1	Incidence reporting	• In the event of a confirmed case, the public health center that first identifies the case reports it to the KDCA and city/province without delay	County/district (*gun/gu*)-level public health centers
		• The case is registered in the COVID-19 Information Management System (https://covid19.kdca.go.kr/)	
		• The case classification is changed from “suspected” to “confirmed” as appropriate	
Stage 2	Basic contact tracing	• Implement a timely investigation to contain and prevent the spread of infection	County/district (*gun/gu*)-level public health centers
		- Personal information, symptoms, pre-existing conditions, and contact with family (cohabitants) and public facilities	
		• Request a bed assignment	
		• Investigate routes of movement in confirmed cases	
		- Request access to CCTV footage, credit card information, visits to multi-purpose facilities, etc.	
Stage 3	In-depth contact tracing	• Classify additional contacts and identify epidemiological associations	City/province
		- Additional investigations, including remembered routes, history of contacts with a confirmed patient (suspicious circumstances), clinical symptoms, route of movement, and contacts	
Stage 4	Contact tracing support	• Update and verify contact tracing	City/province
		- Check confirmed patient’s location information, drug utilization review, credit card records, use of multi-purpose facilities (QR codes), etc.	County/district (*gun/gu*)-level public health centers
			Communicable Diseases Center
Stage 5	Cluster contact tracing	• Conduct a field investigation related to cluster outbreak	City/province
		- Decide appropriate actions for patients, contacts, and environment	County/district (*gun/gu*)-level public health centers
		• Draft a cluster investigation report	Communicable Diseases Center
		- Include results of contact tracing of confirmed patients related to the cluster outbreak and field contact tracing (facility risk assessment, environmental sample results, etc.)	
		• Register cluster investigation report	
		- To be uploaded to the COVID-19 Information Management System within 48 hours after the case investigation by the public health center that identified the cluster outbreak	
Stage 6	Review and circulation	• Assess the situati• Assess the situation and confirm additional disease control measures	City/province
Stage 7	Conclusion of contact tracing	• Decide whether to conclude contact tracing	City/province

COVID-19, coronavirus disease 2019; KDCA, Korean Disease Control and Prevention Agency; CCTV, closed-circuit television. Source from: Incheon Communicable Diseases Center. Novel respiratory infec tious disease epidemiological investigation manual version 1.0; 2021 [6].

**Table 2.** Demographic characteristics of respondents (n=190)

Characteristics	n (%)
Sex	
Male	75 (39.5)
Female	115 (60.5)
Age (yr)	
20-29	61 (32.1)
30-39	82 (43.2)
40-49	33 (17.4)
50-59	12 (6.3)
≥60	2 (1.1)
Affiliation	
Higher-level unit of local government	31 (16.3)
Basic unit of local government	139 (73.2)
Communicable Diseases Center	7 (3.7)
KDCA	13 (6.8)
Position	
Public health doctor	39 (20.5)
Officer	151 (79.5)
Working period (yr)	
<1	90 (47.4)
1-3	81 (42.6)
3-10	13 (6.8)
≥10	6 (3.2)
Location	
Capital area	148 (77.9)
Non-capital area	29 (15.3)
KDCA	13 (6.8)

KDCA, Korean Disease Control and Prevention Agency.

**Table 3.** Results for the usefulness of each contact tracing tool

Variables	Response		
	Positive	Neutral	Negative
Statement from the confirmed patient or facility manager	131 (68.9)	41 (21.6)	18 (9.5)
KI-Pass (QR code)	158 (83.2)	24 (12.6)	8 (4.2)
Credit card records	164 (86.3)	16 (8.4)	10 (5.3)
GPS and CCTV	153 (80.5)	29 (15.3)	8 (4.2)
Safe Call	122 (64.2)	47 (24.7)	21 (11.1)
Sending a disaster message	75 (39.5)	70 (36.8)	45 (23.7)
Handwritten visitor logs	40 (21.1)	61 (32.1)	89 (46.8)

Values are presented as number (%). GPS, global positioning service; CCTV, closed-circuit television.

**Table 4.** Results for other survey items on each contact tracing tool

Variables	Response		
	Positive	Neutral	Negative
Information match rate			
KI-Pass (QR code)	169 (88.9)	18 (9.5)	3 (1.6)
Credit card records	167 (87.9)	22 (11.6)	1 (0.5)
Safe Call	115 (86.5)	71 (12.8)	1 (0.7)
Handwritten visitor logs	30 (15.8)	74 (38.9)	86 (45.3)
Screening test rate			
KI-Pass (QR code)	141 (74.2)	44 (23.2)	5 (2.6)
Credit card records	139 (73.2)	44 (23.2)	7 (3.7)
Safe Call	99 (74.4)	31 (23.3)	3 (2.3)
Handwritten visitor logs	53 (27.9)	90 (47.4)	47 (24.7)
Response rate			
KI-Pass (QR code)	153 (80.5)	34 (17.9)	3 (1.6)
Credit card records	149 (78.4)	37 (19.5)	4 (2.1)
Safe Call	100 (75.2)	33 (24.8)	0 (0.0)
Handwritten visitor logs	56 (29.5)	77 (40.5)	57 (30.0)
Helpfulness			
KI-Pass (QR code)	173 (91.1)	13 (6.8)	4 (2.1)
Credit card records	157 (82.6)	23 (12.1)	10 (5.3)
Safe Call	104 (78.2)	26 (19.5)	3 (2.3)
Handwritten visitor logs	42 (22.1)	86 (45.3)	62 (32.6)

**Table 5.** Results for the order in which information was checked during contact tracing and in case of a mass outbreak

	KI-Pass (QR code)	Credit card records	GPS and CCTV	Safe Call	Handwritten visitor logs
Usually					
1st	30 (15.8)	76 (40.0)	60 (31.6)	8 (4.2)	16 (8.4)
2nd	59 (31.1)	35 (18.4)	57 (30.0)	17 (8.9)	22 (11.6)
3rd	67 (35.3)	33 (17.4)	24 (12.6)	33 (17.4)	33 (17.4)
4th	31 (16.3)	26 (13.7)	27 (14.2)	55 (28.9)	51 (26.8)
5th	3 (1.6)	20 (10.5)	22 (11.6)	77 (40.5)	68 (35.8)
In case of a mass outbreak					
1st	58 (36.7)	45 (28.5)	33 (20.9)	6 (3.8)	16 (10.1)
2nd	46 (29.1)	32 (20.3)	31 (19.6)	28 (17.7)	21 (13.3)
3rd	31 (19.6)	20 (12.7)	24 (15.2)	38 (24.1)	45 (28.5)
4th	18 (11.4)	28 (17.7)	38 (24.1)	41 (25.9)	33 (20.9)
5th	5 (3.2)	33 (20.9)	32 (20.3)	45 (28.5)	43 (27.2)

Values are presented as number (%). GPS, global positioning service; CCTV, closed-circuit television.
