# Supplementary material for: Perceived usefulness of COVID-19 tools for contact tracing among contact tracers in Korea
Source: Epidemiol Health. 2022 Nov 15;44:e2022106. doi: 10.4178/epih.e2022106 (PMC10185965; doi:10.4178/epih.e2022106)
Supplement: Supplementary Material 1. [file epih-44-e2022106-Supplementary-1.pdf]

Supplementary materials

Perceived usefulness of COVID-19 tools for contact tracing among contact tracers in Korea

Contents

Supplementary information 1. Survey in Korean Language

Supplementary information 1. Survey in Korean Language

코로나19 확진자 역학조사 시, 출입명부 사용에 관한 설문

본 설문에 참여하여 수집되는 개인정보(성별, 연령대)는
본 연구에만 사용되며 통계법 제 33조에 의하여 개인의 비밀은 반드시 보장됩니다.
연구 종료 후에도 귀하의 신원을 파악할 수 있는 기록은 비밀 상태로 유지될 것입니다.

1. 귀하의 성별은 무엇입니까?
① 남
② 여

2. 귀하의 연령대는 무엇입니까?
① 20대
② 30대
③ 40대
④ 50대
⑤ 60대 이상

3. 귀하의 소속은 어디입니까?
① 시군구 (기초지자체)
② 시도 (광역지자체)
③ 감염병관리지원단
④ 질병관리청
⑤ 기타

4. 귀하의 감염병 관련 근무 경력은 몇 년 입니까?
① 1년 미만
② 1년 이상 ~ 3년 미만
③ 3년 이상 ~ 10년 미만
④ 10년 이상

5. 귀하의 업무 담당 지역은 어느 곳입니까?
① 서울
② 부산
③ 대구
④ 인천
⑤ 경기
⑥ 광주
⑦ 대전
⑧ 울산
⑨ 세종
⑩ 강원
⑪ 충북
⑫ 충남
⑬ 전북
⑭ 전남
⑮ 경북
⑯ 경남
⑰ 제주
⑱ 기타

각 역학조사 방법의 활용성

다음은 코로나19 확진자 방문 시설의 접촉자 파악 시, 아래 역학조사 방법의 활용성에 대한 설문입니다.

1. [확진자 또는 시설관리자의 진술]의 코로나19 확진자 방문 시설의 접촉자 파악 시 활용성
활용성 매우 낮음 ① ② ③ ④ ⑤ 활용성 매우 높음

2. 확진자 또는 시설관리자의 진술에 대해 위와 같이 생각하신 이유

3. [GPS 및 CCTV정보]의 코로나19 확진자 방문 시설의 접촉자 파악 시 활용성
활용성 매우 낮음 ① ② ③ ④ ⑤ 활용성 매우 높음

4. GPS 및 CCTV정보에 대해 위와 같이 생각하신 이유

5. [카드결제정보]의 코로나19 확진자 방문 시설의 접촉자 파악 시 활용성
활용성 매우 낮음 ① ② ③ ④ ⑤ 활용성 매우 높음

6. 카드결제정보에 대해 위와 같이 생각하신 이유

7. [방문시설 수기명부]의 코로나19 확진자 방문 시설의 접촉자 파악 시 활용성
활용성 매우 낮음 ① ② ③ ④ ⑤ 활용성 매우 높음

8. 방문시설 수기명부에 대해 위와 같이 생각하신 이유

9. [방문시설 KI-PASS (QR코드)]의 코로나19 확진자 방문 시설의 접촉자 파악 시 활용성
활용성 매우 낮음 ① ② ③ ④ ⑤ 활용성 매우 높음

10. 방문시설 KI-PASS (QR코드)에 대해 위와 같이 생각하신 이유

11. [방문시설 안심콜]의 코로나19 확진자 방문 시설의 접촉자 파악 시 활용성
활용성 매우 낮음 ① ② ③ ④ ⑤ 활용성 매우 높음

12. 방문시설 안심콜에 대해 위와 같이 생각하신 이유

13. [재난문자 발송]의 코로나19 확진자 방문 시설의 접촉자 파악 시 활용성
활용성 매우 낮음 ① ② ③ ④ ⑤ 활용성 매우 높음

14. 재난문자 발송에 대해 위와 같이 생각하신 이유

통상적인 접촉자 파악 순서

귀하께서 코로나19 확진자의 역학조사 시, 역학조사서 작성 후 접촉자 파악을 위해 통상적으로 확인하는 정보의 순서를 선택하여 주십시오.

1. 역학조사서 작성 후 접촉자 파악을 위해 [1순위]로 확인하는 정보

① 카드결제정보
② 방문시설 수기명부
③ 방문시설 KI-PASS (QR코드)
④ 방문시설 안심콜
⑤ GPS 및 CCTV 정보

2. [2순위]로 확인하는 정보

① 카드결제정보
② 방문시설 수기명부
③ 방문시설 KI-PASS (QR코드)
④ 방문시설 안심콜
⑤ GPS 및 CCTV 정보

3. [3순위]로 확인하는 정보

① 카드결제정보
② 방문시설 수기명부
③ 방문시설 KI-PASS (QR코드)
④ 방문시설 안심콜
⑤ GPS 및 CCTV 정보

4. [4순위]로 확인하는 정보

① 카드결제정보
② 방문시설 수기명부
③ 방문시설 KI-PASS (QR코드)
④ 방문시설 안심콜
⑤ GPS 및 CCTV 정보

대유행 시, 접촉자 파악 순서

귀하께서 전국적인 '대유행' 시, 역학조사서 작성 후 접촉자 파악을 위해 확인하는 정보의 순서를 선택하여 주십시오.

1. '대유행' 시, [1순위]로 확인하는 정보

① 카드결제정보
② 방문시설 수기명부
③ 방문시설 KI-PASS (QR코드)
④ 방문시설 안심콜
⑤ GPS 및 CCTV 정보

2. [2순위]로 확인하는 정보

① 카드결제정보
② 방문시설 수기명부
③ 방문시설 KI-PASS (QR코드)
④ 방문시설 안심콜
⑤ GPS 및 CCTV 정보

3. [3순위]로 확인하는 정보

① 카드결제정보
② 방문시설 수기명부
③ 방문시설 KI-PASS (QR코드)
④ 방문시설 안심콜
⑤ GPS 및 CCTV 정보

4. [4순위]로 확인하는 정보

① 카드결제정보
② 방문시설 수기명부
③ 방문시설 KI-PASS (QR코드)
④ 방문시설 안심콜
⑤ GPS 및 CCTV 정보

5. 귀하께서는 코로나19의 '대유행' 시, 그렇지 않을 때와 비교하여 역학조사 방법의 가장 큰 차이점은 무엇이라고 생각하십니까?

KI-PASS(QR코드)의 사용

다음은 코로나19 확진자의 역학조사 시, 방문 시설의 KI-PASS(QR코드)의 사용에 관한 설문입니다.

1. 확진자 방문 시설의 KI-PASS(QR코드)정보가 실제 출입자 정보와 어느 정도 일치한다고 생각하십니까?

전혀 일치하지 않음 ① ② ③ ④ ⑤ 매우 일치함

2. 역학조사 시, KI-PASS(QR코드)와 실제 출입자 정보의 일치도에 대한 자유로운 의견 부탁드립니다.

3. 확진자 방문 시설의 KI-PASS(QR코드)정보로 접촉자 전화 연결 시, 실제 선별검사를 실시한 접촉자는 어느 정도라고 생각하십니까?

전혀 실시하지 않음 ① ② ③ ④ ⑤ 모두 실시함

4. 확진자 방문 시설의 KI-PASS(QR코드)정보로 접촉자 전화 연결 시, 실제 연락에 응답하였던 접촉자는 어느 정도라고 생각하십니까?

모두 연락이 가능함 ① ② ③ ④ ⑤ 모두 연락이 불가능함

5. 확진자 방문 시설의 KI-PASS(QR코드)정보로 접촉자 전화 연결에 대한 자유로운 의견 부탁드립니다.

6. 그렇다면, KI-PASS(QR코드)가 역학조사 전반에 걸쳐 얼마나 도움이 된다고 생각하십니까?

전혀 도움이 되지 않음 ① ② ③ ④ ⑤ 매우 도움이 됨

7. 역학조사 시, KI-PASS(QR코드) 사용에 대한 자유로운 의견 부탁드립니다.

카드결제정보의 사용

다음은 코로나19 확진자의 역학조사 시, 방문 시설의 카드결제정보의 사용에 관한 설문입니다.

1. 확진자 방문 시설의 카드결제정보가 실제 출입자 정보와 어느 정도 일치한다고 생각하십니까?
전혀 일치하지 않음 ① ② ③ ④ ⑤ 매우 일치함

2. 역학조사 시, 카드결제정보와 실제 출입자 정보의 일치도에 대한 자유로운 의견 부탁드립니다.

3. 확진자 방문 시설의 카드결제정보로 접촉자 전화 연결 시, 실제 선별검사를 실시한 접촉자의 비율은 어느 정도라고 생각하십니까?
전혀 실시하지 않음 ① ② ③ ④ ⑤ 모두 실시함

4. 확진자 방문 시설의 카드결제정보로 접촉자 전화 연결 시, 실제 연락에 응답하였던 접촉자는 어느 정도라고 생각하십니까?
모두 연락이 가능함 ① ② ③ ④ ⑤ 모두 연락이 불가능함

5. 확진자 방문 시설의 카드결제정보로 접촉자 전화 연결에 대한 자유로운 의견 부탁드립니다.

6. 그렇다면, 카드결제정보가 역학조사 전반에 걸쳐 얼마나 도움이 된다고 생각하십니까?
전혀 도움이 되지 않음 ① ② ③ ④ ⑤ 매우 도움이 됨
7. 역학조사 시, 카드결제정보 사용에 대한 자유로운 의견 부탁드립니다.

수기명부의 사용

다음은 코로나19 확진자의 역학조사 시, 방문 시설의 수기명부의 사용에 관한 설문입니다.

1. 확진자 방문 시설의 수기명부가 실제 출입자 정보와 어느 정도 일치한다고 생각하십니까?
전혀 일치하지 않음 ① ② ③ ④ ⑤ 매우 일치함

2. 역학조사 시, 수기명부와 실제 출입자 정보의 일치도에 대한 자유로운 의견 부탁드립니다.

3. 확진자 방문 시설의 수기명부로 접촉자 전화 연결 시, 실제 선별검사를 실시한 접촉자의 비율은 어느 정도라고 생각하십니까?
전혀 실시하지 않음 ① ② ③ ④ ⑤ 모두 실시함

4. 확진자 방문 시설의 수기명부로 접촉자 전화 연결 시, 실제 연락에 응답하였던 접촉자는 어느 정도라고 생각하십니까?
모두 연락이 가능함 ① ② ③ ④ ⑤ 모두 연락이 불가능함

5. 확진자 방문 시설의 수기명부로 접촉자 전화 연결에 대한 자유로운 의견 부탁드립니다.

6. 그렇다면, 수기명부가 역학조사 전반에 걸쳐 얼마나 도움이 된다고 생각하십니까?
전혀 도움이 되지 않음 ① ② ③ ④ ⑤ 매우 도움이 됨

7. 역학조사 시, 수기명부 사용에 대한 자유로운 의견 부탁드립니다.

안심콜의 사용

다음은 코로나19 확진자의 역학조사 시, 방문 시설의 안심콜 출입관리 시스템 사용에 관한 설문입니다.

1. 귀하께서는 코로나19 확진자의 역학조사 시, 안심콜의 정보를 사용해보신 적이 있으십니까?
① 예
② 아니요 [다음 설문으로 이동]

안심콜의 사용 [안심콜 경험자만 응답]

다음은 코로나19 확진자의 역학조사 시, 방문 시설의 안심콜 출입관리 시스템 사용에 관한 설문입니다.

1. 확진자 방문 시설의 안심콜이 실제 출입자 정보와 어느 정도 일치한다고 생각하십니까?
전혀 일치하지 않음 ① ② ③ ④ ⑤ 매우 일치함

2. 역학조사 시, 안심콜과 실제 출입자 정보의 일치도에 대한 자유로운 의견 부탁드립니다.

3. 확진자 방문 시설의 안심콜 정보로 접촉자 전화 연결 시, 실제 선별검사를 실시한 접촉자의 비율은 어느 정도라고 생각하십니까?
전혀 실시하지 않음 ① ② ③ ④ ⑤ 모두 실시함

4. 확진자 방문 시설의 안심콜 정보로 접촉자 전화 연결 시, 실제 연락에 응답하였던 접촉자는 어느 정도라고 생각하십니까?
모두 연락이 가능함 ① ② ③ ④ ⑤ 모두 연락이 불가능함

5. 확진자 방문 시설의 안심콜 정보로 접촉자 전화 연결에 대한 자유로운 의견 부탁드립니다.

6. 그렇다면, 안심콜이 역학조사 전반에 걸쳐 얼마나 도움이 된다고 생각하십니까?
전혀 도움이 되지 않음 ① ② ③ ④ ⑤ 매우 도움이 됨

7. 귀하께서는 해당지역에 안심콜이 도입된 후, 역학조사 시 안심콜의 사용빈도 변화에 대해 생각하십니까?
① 안심콜 도입 후 사용빈도 증가
② 안심콜 도입 전과 후 사용빈도 변화 없음

8. 역학조사 시, 안심콜 사용에 대한 자유로운 의견 부탁드립니다.

담당 지역에 가장 적합한 역학조사 방법

1. 귀하의 업무 담당 지역은 아래 문항에 대하여 어느 역학조사 방법이 가장 적합하다고 생각하십니까?

	수기명부	KI-PASS (QR코드)	카드결제 정보	안심콜	GPS 및 CCTV 정보	제주안심 코드	클린강원 패스포트
역학조사의 편의성	○	○	○	○	○	○	○
시설 출입자의 편의성	○	○	○	○	○	○	○
시설 관리자의 정확한 출입자 관리	○	○	○	○	○	○	○
